# Circulating Autoantibodies against the Apolipoprotein B-100 Peptides p45 and p210 in Relation to the Occurrence of Carotid Plaques in 64-Year-Old Women

**DOI:** 10.1371/journal.pone.0120744

**Published:** 2015-03-13

**Authors:** Björn Fagerberg, Ulrica Prahl Gullberg, Ragnar Alm, Jan Nilsson, Gunilla Nordin Fredrikson

**Affiliations:** 1 Wallenberg Laboratory for Cardiovascular Research, Sahlgrenska Academy, University of Gothenburg, Gothenburg, Sweden; 2 Department of Clinical Sciences, Skåne University Hospital Malmö, Lund University, Malmö, Sweden; University of Amsterdam Academic Medical Center, NETHERLANDS

## Abstract

**Objectives:**

Immune responses against oxidized low density lipoprotein (LDL) play a key role in atherosclerosis. Previous studies have indicated inverse associations between autoantibodies to epitopes in oxidized LDL and cardiovascular disease. In this study we investigated the associations between autoantibodies against the apolipoprotein B-100 (apoB-100) peptides p45 and p210 and occurrence of carotid plaques.

**Design:**

The study cohort consisted of a population-based sample of 64-year-old women with varying degrees of glucose tolerance (n=594). To identify and record the occurrence of carotid atherosclerotic plaques ultrasonography was used. Measurements of plasma IgM and IgG autoantibodies against the native and malondialdehyde (MDA)-modified apoB-100 peptides p45 and p210 were performed by ELISA.

**Results:**

Women with carotid plaques were found to have lower levels of IgM MDA-p210 autoantibodies compared to plaque-free women. The number of carotid plaques in each subject and the total carotid plaque area correlated inversely with IgM MDA-p210 levels (r=-0.11, P=0.009 and r=-0.11, P=0.013, respectively). Furthermore, levels of IgM MDA-p210 above the lowest tertile were associated with an odds ratio of 0.55 (95% CI 0.38-0.79, P=0.001) for occurrence of carotid plaques, independently of other risk markers and statin treatment. Associations between apo-B100 peptide autoantibodies and cardiovascular risk factors were generally weak but subjects with impaired glucose tolerance had higher levels of IgM against MDA-p210.

**Conclusion:**

The present study demonstrates that high levels of IgM against MDA-p210 are associated with less severe carotid disease in women. These findings provide additional support for a role of immune responses against oxidized LDL in cardiovascular disease.

## Introduction

The role of immune responses against modified self-antigens as oxidized low density lipoprotein (LDL) in atherosclerosis has been the focus of several studies during the last decades [1, 2]. Both plasma and plaques from humans and hypercholesterolaemic animals have revealed presence of autoantibodies and T cells recognizing oxidized LDL [3–6]. Experimental animal studies have suggested that Th1 immune responses against self-antigens modified by hypercholesterolemia play an important role in driving atherosclerosis [1, 7, 8]. However, immunization of hypercholesterolaemic animals with oxidized LDL or peptides of the major protein in LDL, apolipoprotein B-100 (apoB-100), resulted in reduced atherosclerosis development, indicating that also athero-protective immune responses exist [9–13]. During oxidation of LDL, the apoB-100 becomes fragmented and aldehyde-modified, making the particle an immunogenic target [3, 14]. We have previously identified several native and aldehyde-modified apoB-100 peptides that are recognized by autoantibodies in human plasma [15]. Immune responses against the apoB-100 amino acids 661–680 (p45) and 3136–3155 (p210) have been found to be of particular interest. Individuals with high levels of IgG against the native forms of p45 [16] and p210 [17] have a lower risk of development of acute myocardial infarction. An inverse relation between IgG against native p210 and the severity of coronary atherosclerosis has also been demonstrated [17].

Type 2 diabetes is accompanied by high incidence of both subclinical and clinical atherosclerotic disease [18, 19]. It is also associated with increased vascular oxidative stress and presence of LDL with increased susceptibility to oxidation [20]. This suggests that immune responses to modified antigens in LDL may be of particular importance in the development of diabetic macrovascular complications. We have recently shown that high levels of IgG and IgM autoantibodies against native p45 and p210 were associated with less coronary calcification and a lower risk of progression of coronary disease in patients with type 2 diabetes [21]. In addition, we have demonstrated that high levels of IgM against methylglyoxal (MGO)-apoB-100 are associated with less severe and a lower risk of progression of coronary disease in subjects with type 2 diabetes [22].

The primary aim of the present study was to examine if any of the p45 or p210 autoantibodies were associated with the occurrence and size of atherosclerotic plaques in the carotid arteries in a population cohort of 64-year-old women with various degrees of glucose tolerance. We also explored the associations between the antibody levels and cardiovascular risk factors.

## Material and Methods

### Study cohort

The Diabetes, Impaired glucose tolerance in Women and Atherosclerosis (DIWA) study was designed and powered to examine subclinical atherosclerosis in the carotid arteries in a population cohort of 64-year-old women with various degrees of glucose tolerance [23–25]. Briefly, 2595 women identified through the County Register were contacted and screened with an oral glucose tolerance test (OGTT). Diabetes and impaired glucose tolerance (IGT) were defined according to the WHO classification [26]. A stratified randomization procedure was used to include similarly-sized groups of subjects with diabetes, impaired and normal glucose tolerance (n = 213/191/190, respectively) in a nested case-control design. The exclusion criteria were malignant or inflammatory disease, severe psychiatric disorders, or other circumstances making participation not feasible.

The participants completed questionnaires regarding life style factors, previous and current diseases and medication (S1 Table). Anthropometrical measurements were performed, blood pressure was recorded and blood samples were drawn when the participants had fasted overnight. Plasma and serum samples were stored in -70°C. Measurements of serum concentrations of LDL cholesterol, HDL cholesterol, triglycerides, apolipoproteins A-I and B, fasting glucose, blood HbA1c and high sensitive C-reactive protein (hsCRP) were done as previously described [23, 24, 27]. Ultrasound examination of both carotid arteries was performed.

### Ethics Statement

The subjects received both written and oral information before they gave their written consent to participate. The study was carried out in accordance with the Declaration of Helsinki and was approved by the Ethics Committee at Sahlgrenska University Hospital in Gothenburg (S 286–01).

### Ultrasound examination

As previously described, examinations were performed with an ultrasound scanner equipped with a linear 8L5-MHz transducer (Sequoia 512, Siemens, Mountain View, CA). An electrocardiographic signal (lead II) was simultaneously recorded to synchronize image capture to the peak of the R wave to minimize variability during the cardiac cycle. To identify and record the occurrence of atherosclerotic plaques, carotid arteries were scanned from the distal part of the common carotid artery to 10 mm into the external and internal carotid arteries. A sequence of real-time images (real-motion loop) was captured and saved from the position yielding the best visibility of the plaque (i.e., the largest cross-sectional area in a longitudinal transaxial view, as judged visually) and saved digitally. A plaque was defined as a distinct area with an intima-media thickness 50% greater than neighbouring sites, as judged visually. Total plaque area was obtained by a semi-automated program [27]. From the real-motion loop, an R-wave-triggered longitudinal image for each plaque was saved digitally.

### Determination of p45 and p210 autoantibodies

Peptides corresponding to the amino acids from 661 to 680 (p45; IEIGLEGKGFEPTLEALFGK) and amino acids 3136–3155 (p210; KTTKQSFDLSVKAQYKKNKH) of human apoB-100 were synthesized (KJ Ross Petersen AS, Horsholm, Denmark) and used in ELISA. The peptides were modified by 0.5 M MDA for 3 h at 37°C and dialyzed against PBS containing 1 mM EDTA as described [15]. Native and MDA-modified peptides diluted in PBS pH 7.4 (20 μg/ml) were absorbed to microtiter plate wells (Nunc MaxiSorp, Nunc, Roskilde, Denmark) in an overnight incubation at 4°C. After washing with PBS containing 0.01% Tween-20 (PBS-T) the coated plates were blocked with SuperBlock in TBS (Pierce, Rockford, Illinois) for 30 min at room temperature (RT) followed by an incubation of test plasma, diluted 1/100 in TBS-0.01% Tween-20 (TBS-T) for 2 h at RT and overnight at 4°C. After rinsing, deposition of autoantibodies directed to the peptide was detected using biotinylated rabbit anti-human IgM (ICN, Biomedicals, Inc., Aurora, OH) or IgG antibodies (Dako A/S, Glostrup, Denmark) appropriately diluted in TBS-T. After another incubation for 2 h at RT the plates were washed and the bound biotinylated antibodies detected by alkaline phosphatase conjugated streptavidin (Sigma), incubated for 2 h at RT. The colour reaction was developed by using phosphatase substrate kit (Pierce) and the absorbance at 405 nm was measured after 1 h of incubation at RT. Data regarding the specificity and variability of the antibody ELISA have been published previously [15, 16].

### Statistics

SPSS (18.0) was used for the statistical analysis. The results are presented as mean (standard deviation) or median (IQR, interquartile range) for skewed variables and as proportions when appropriate. Mann-Whitney and chi-square tests were used for comparison between groups. Spearman rank correlation test for univariate correlations and logistic regression analysis for analysis of independent covariates to occurrence of plaques. Skewed variables were log transformed. In the logistic regression analyses carotid plaque occurrence was the dependent variable and two models were used to adjust for confounders. Model 1 included autoantibody levels and prevalent diabetes, as being the only potential confounders showing univariate associations with both carotid plaque occurrence and concentrations of the autoantibody under analysis. Model 2 included additional full adjustment for known cardiovascular risk factors (smoking, apolipoproteinB/A-I ratio, blood pressure, HbA1c and treatment with statins). *P*<0.05 (two-sided) was regarded as statistically significant.

## Results

**Fig 1:**
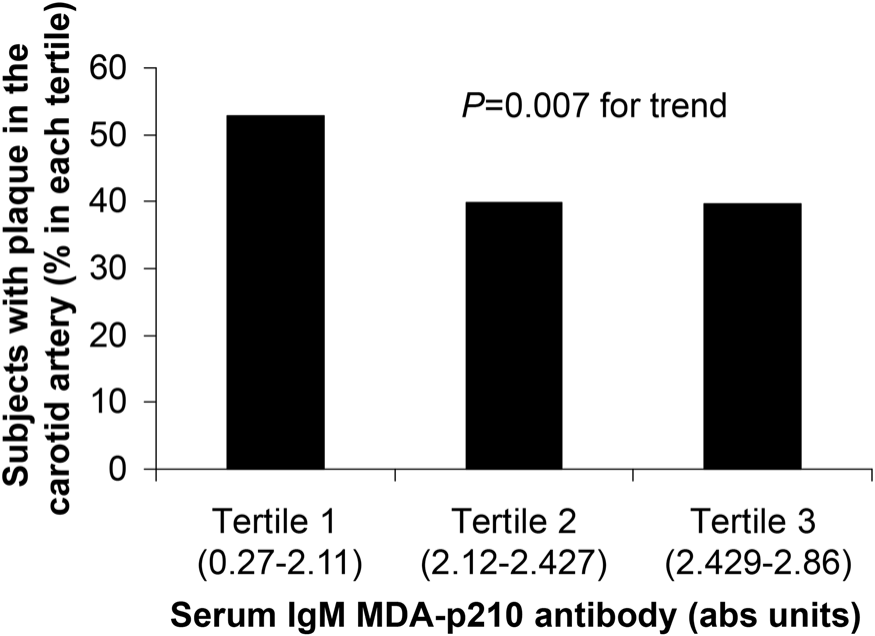
Relation between IgM MDA-p210 autoantibody levels and presence of carotid plaques. The figure shows the proportion of 64-year-old women with prevalent atherosclerotic plaques in the carotid arteries in each tertile of serum concentrations of IgM MDA-p210 autoantibodies. Chi-square test was used for comparison between groups. Abs units, absorbance units at 405 nm.

As shown in Table 1, in comparison with plaque-free women, those with carotid plaques were characterized by a higher incidence of diabetes, higher blood levels of HbA1c, fewer never-smokers and more women with statin treatment. Those with plaques also had lower plasma levels of IgM autoantibodies against MDA-p210 (Table 2 and Fig. 1), whereas none of the other antibodies differed between the groups.

**Table 1:** Characteristics of 64-year old women in relation to occurrence of plaques in the carotid arteries.

	No plaque(n = 332)	Plaque(n = 262)
Smoking, n(%)		
Never	167(50.3)	94(35,9)***
Previous	117(35.2)	93(35.5)
Current	48(14.5)	75(28.6)
Statin treatment, n(%)	30(9.3)	46(17.7)**
BMI, (mean, SD)	28.0(4.7)	27.6(4.5)
Waist, cm (mean, SD)	93.3(12.2)	93.4(11.9)
Systolic BP, mm Hg (mean, SD)	143 (18)	146(19)
Diastolic BP, mm Hg (mean, SD)	81(8)	80(9)
Diabetes, n(%)	107 (32.2)	106 (40.5)*
IGT, n(%)	116(34.9)	75(28.6)
Fasting glucose, mmol/L	5.24(1.35)	5.37(1.67)
HbA1c, % (median, IQR)	4.7(0.6)	4.8(1.1)*
LDL, mmol/L (mean, SD)	3.54(0.93)	3.49(1.04)
HDL, mmol/L (mean, SD)	1.63(0.44)	1.63(0.55)
Triglycerides, mmol/L (median, IQR)	1.26(0.82)	1.32(0.87)
ApoB/ApoA-1 (mean, SD)	0.75(0.22)	0.75(0.22)
CRP (median, IQR)	1.45(2.60)	1.53(2.50)

BMI, body mass index; BP, blood pressure; IGT, impaired glucose tolerance.

*p<0.05

**p<0.01

***p<0.001

**Table 2:** Autoantibodies to the apoB-100 peptides p45 and p210 in 64-year-old women in relation to occurrence of atherosclerotic plaques in the carotid arteries.

		No plaque(n = 332)	Plaque(n = 262)
Ab to native p45	IgG	0.19 (0.30)	0.20 (0.27)
	IgM	0.16 (0.26)	0.19 (0.26)
Ab to native p210	IgG	1.15 (0.50)	1.16 (0.51)
	IgM	1.16 (0.55)	1.14 (0.61)
Ab to MDA-p45	IgG	0.81 (0.52)	0.78 (0.46)
	IgM	1.06 (0.70)	1.04 (0.69)
Ab to MDA-p210	IgG	2.44 (0.40)	2.40 (0.42)
	IgM	2.33 (0.42)	2.23 (0.46)**

Ab, antibodies; Values given as absorbance units at 405 nm; Values are median (IQR)

**P = 0.009

The number of carotid plaques in each subject (ranging from 0 to 5) and the total carotid plaque area (ranging from 0–157 mm^2^) correlated inversely with IgM MDA-p210 autoantibody levels (r = -0.11, *P* = 0.009 and r = -0.11, *P* = 0.013, respectively).

### Associations between autoantibodies and cardiovascular risk factors

The autoantibodies recognizing the apoB-100 peptides (native-p45, MDA-p45, native-p210 and MDA-p210) in general showed positive correlations to each other. Both the IgM and the IgG autoantibodies presented significant correlations to most of the other IgM and IgG autoantibodies against the native and the MDA-modified peptides p45 and p210 (r-values between 0.125 and 0.605 and with *P*-values <0.05 or <0.01). The exceptions were that IgG native-p45 showed no correlation to IgM MDA-p45, IgG native-p210, IgM native-p210 or IgM MDA-p210 and also no correlation was detected between IgG MDA-p45 and IgM MDA-p210.

Several autoantibodies were associated with cardiovascular risk factors and treatment with statins (Table 3 and S2–S3 Tables). However, smoking history showed no statistically significant associations with the autoantibody levels (data not shown). IgG native p210 autoantibody levels correlated negatively with blood pressure, HbA1c, C-reactive protein (S2 Table) and statin treatment (S3 Table). IgM native p210 antibody levels showed similar correlations with blood pressure and statin treatment. IgG native p45 antibody levels correlated negatively with apolipoprotein B concentrations (S2 Table), whereas IgM native p45 antibody levels were lower in those with, than with no diabetes (Table 3). IgM MDA-p45 antibody levels correlated negatively with systolic blood pressure, diabetes and statin treatment (Table 3 and S2–S3 Tables). The corresponding IgG antibody concentrations were lower in women with, than without statin treatment (S3 Table). IgG MDA-p210 antibody levels correlated negatively with fasting blood glucose (S2 Table). IgM MDA-p210 differed from the other antibodies as diabetes in comparison with normal glucose tolerance was associated with a higher autoantibody concentration (Table 3).

**Table 3:** Autoantibodies to the apoB-100 peptides p45 and p210 in 64-year-old women with diabetes, impaired (IGT) and normal (NGT) glucose tolerance.

		Diabetes	IGT	NGT	*P*-value for trend
Ab to native p45	IgG	0.21 (0.27)	0.18 (0.32)	0.18 (0.26)	1.00
	IgM	0.16 (0.24)	0.17 (0.29)	0.21 (0.27)	0.006
Ab to native p210	IgG	1.14 (0.64)	1.14 (0.46)	1.22 (0.51)	0.24
	IgM	1.17 (0.58)	1.17 (0.60)	1.14 0.52)	0.48
Ab to MDA-p45	IgG	0.80 (0.53)	0.72 (0.52)	0.85 (0.48)	0.12
	IgM	1.00 (0.72)	0.97 (0.65)	1.16 (0.64)	0.014
Ab to MDA-p210	IgG	2.39 (0.38)	2.44 (0.38)	2.44 (0.43)	0.092
	IgM	2.30 (0.46)	2.34 (0.34)	2.16 (0.50)	0.030

Ab, antibodies; Values given as absorbance units at 405 nm; Values are median (IQR).

### Multivariate analyses

To further study the association between IgM MDA-p210 autoantibody levels and presence of carotid plaques we divided the study cohort into IgM MDA-p210 tertiles. The 1–3 tertiles of the IgM MDA-p210 autoantibody concentrations consisted of 198, 199 and 197 women, and plaques were found in 53% (n = 105), 40% (n = 79) and 40% (n = 78), respectively (see Fig. 1). The upper limit of the the lowest tertile of MDA-p210 IgM (<2.12 abs units), was used to divide the cohort into those below and above this cut-off. A logistic regression analysis was performed with occurrence of plaque as dependent variable and the occurrence of diabetes as the only potential confounder that was associated with both plaque occurrence and IgM MDA-p210 antibody concentrations (Model 1). As shown in Table 4 the autoantibody levels of MDA-p210 IgM above that of the lowest tertile were associated with an odds ratio of 0.58 (95% C.I. 0.41–0.82, *P* = 0.002) for occurrence of carotid plaques. In a second analysis including all usual risk factors for cardiovascular disease as well as treatment with statins the result remained with an odds ratio of 0.55 (95% C.I. 0.38–0.79, *P* = 0.001). Smoking, systolic blood pressure and statin treatment remained also independently associated with carotid atherosclerosis (Table 4, Model 2).

**Table 4:** Logistic regression with presence of carotid plaque as dependent variable in 594 women, 64 years of age.

Variable	Model 1		Model 2	
	OR (95% CI)	*P*	OR (95% CI)	*P*
IgM MDA-p210 (0 < 2.11 abs units^#^, 1 = above this level)	0.58 (0.41–0.82)	0.002	0.55 (0.38–0.79)	0.001
Diabetes (0 = no, 1 = yes)	1.47 (1.04–2.06)	0.027	1.01 (0.65–1.59)	0.95
Smoking history				
Never smoker			1	
Previous smoker			1.45 (0.99–2.13)	0.058
Current smoker			2.79 (1.76–4.42)	<0.001
Statin treatment (0 = no, 1 = yes)			1.86 (1.09–3.17)	0.024
HbA1c, %			1.14 (0.94–1.40)	0.19
Systolic blood pressure, mm Hg			1.01 (1.00–1.02)	0.019
Apolipoprotein B/A-I			0.93 (0.42–2.11)	0.87

CI, confidence interval; *P*, p-value.

^#^Abs units, absorbance units at 405 nm. The chosen cut-off corresponds to the upper level of the first tertile of IgM MDA-p210.

## Discussion

In the present study we found that circulating levels of IgM autoantibodies against MDA-p210 were inversely associated with occurrence of carotid plaques. Autoantibody levels above that of the lowest tertile showed an odds ratio of 0.55 (95% C.I. 0.38–0.79) for occurrence of any plaque in the carotid arteries, independently of other risk markers and treatment with statins. The number of carotid plaques and the total carotid plaque area were also inversely correlated with the levels of IgM MDA-p210.

The cohort in the present study is characterized by a stratified randomization procedure, resulting in an enrichment of subjects with diabetes and impaired glucose tolerance and increased risk of elevated inflammatory biomarkers and carotid atherosclerosis [24, 25, 28]. Our results demonstrated inverse correlations between both IgG and IgM autoantibodies recognizing p45 and p210 and cardiovascular risk factors as blood pressure, fasting blood glucose and diabetes. This is in agreement with previous findings showing a negative association between IgG titers to oxidized LDL and markers of glucose metabolism and that low levels of IgG against oxidized LDL are a risk factor for type 2 diabetes [29]. In addition, it has been reported that women with hypertension have lower levels of IgG autoantibodies recognizing oxidized LDL [30]. However, the present IgM MDA-p210 autoantibody showing an association with lower risk of occurrence of carotid plaques was surprisingly found to be at higher concentrations in diabetic patients than in individuals with normal glucose tolerance. It has previously been demonstrated that IgM autoantibodies to oxidized LDL were significantly reduced by both atorvastatin and pravastatin treatment [31], in accordance with our findings that women on statin treatment had lower levels of some of the apoB-100 autoantibodies. Furthermore, patients with multi-vessel coronary artery disease treated with statins was found to have lower levels of IgM oxidized LDL autoantibodies than those without multi-vessel coronary artery disease and statin treatment [32]. This is in line with our observations that statin treatment was associated with carotid disease and low levels of IgM MDA-p210 with increased risk of occurrence of carotid plaques.

Previous reports studying the associations between autoantibodies to oxidized LDL and cardiovascular disease have provided inconsistent results [33–36]. One explanation may be the technical difficulties in standardising ELISAs based on such a complex antigen as oxidized LDL. Oxidized LDL is poorly defined as antigen and neo-eptiopes are continually formed and degraded during the oxidation process. Using better characterized antigens, such as native and MDA-modified peptides of apoB-100, may circumvent this problem [15]. Accordingly, measurements of autoantibodies to apoB-100 peptides may have an advantage over measurements of oxidized LDL autoantibodies. Our previous studies measuring autoantibodies against different apoB-100 peptides have consistently demonstrated inverse associations between such antibodies and the severity of arterial disease in a number of studies [16, 17, 21, 22, 37, 38]. Other reasons could be that the oxidized LDL autoantibody measurement has included either the IgG or the IgM isotype, and that heterogeneous clinical materials have been used [33, 36]. Boullier et al. showed similar levels of IgG-LDL immune complexes in patients with coronary artery disease and controls, and Festa et al. decreased anti-oxidized LDL IgG in patients with long duration of type 1 diabetes and high HbA1c levels compared to controls, whereas Karvonen et al, demonstrated an inverse relation between anti-oxidized LDL IgM and carotid artery atherosclerosis [33, 34, 36]. Interestingly, some previous studies have found divergent associations between IgM and IgG autoantibodies against oxidized LDL, intima-media thickness and coronary artery disease [39, 40]. However, most recently a larger study including 748 cases and 1723 controls showed that IgG and IgM autoantibodies to MDA-LDL and apoB immune complexes were not independent predictors of coronary artery disease [41].

We have previously shown that post-infarction patients have significantly lower IgG autoantibodies to native p210 compared to controls [17]. The same study also demonstrated an inverse relation between IgG against native p210 and the severity of coronary atherosclerosis. In addition, a prospective study showed that cases developing acute cardiac events have significantly lower IgG levels against native p45 than controls and that low levels of MDA-p45 IgG were associated with a higher degree of carotid stenosis [16]. Moreover, another study including patients with type 2 diabetes demonstrated that high levels of IgM and IgG autoantibodies to the peptides p45 and p210 were associated with less coronary calcification [21]. New findings in the present study revealed that high levels of the IgM MDA-p210 autoantibody associated with fewer occurrences of carotid plaques as well as that number of carotid plaques in each subject and the total carotid plaque area correlated negatively with this autoantibody. Previously an association between IgM autoantibodies against MDA-p210 and a more stable plaque phenotype as well as lower intima-media thickness or slower intima-media thickness progression have been described [37, 38]. Taken together, in most studies IgG and IgM autoantibodies to specific epitopes in apoB-100, seem to indicate a protective role of the autoantibodies. Existence of an atheroprotective immune response against epitopes in apoB-100 is supported by experimental studies, where mice have been immunized with native and MDA-modified apoB-100 peptides resulting in reduced atherosclerosis [11, 12]. Moreover, treatment of mice with human recombinant IgG specific for the MDA-p45 epitope also reduced aortic plaque area and plaque inflammation [42].

In line with the present findings, it has previously been shown some evidence that higher levels of IgM autoantibodies to MDA-LDL can reduce the proatherogenic effect of some oxidative markers suggesting an atheroprotective role of IgM autoantibodies [41]. The protective function of IgM is also supported by the development of accelerated atherosclerosis in IgM deficient mice [43]. In patients with type 2 diabetes high levels of IgM autoantibodies against AGE-modified self-antigens such as MGO-modified apoB-100 were associated with a less severe coronary disease [22]. These IgM autoantibodies were found to be significantly higher in females than in males. Interestingly, another study also demonstrated higher levels of IgM, this time against oxidized LDL, in women than in men and women also had less atherosclerosis [44]. In the present study only women were included, but all together the findings may reflect a gender difference that women are at lower cardiovascular risk because of higher autoantibody levels against antigens important in the development of cardiovascular diseases.

One limitation of the present study is the cross-sectional design, since it does not give the opportunity to determine any causal relationship between the apoB-100 peptide autoantibodies and the atherosclerotic disease process. Moreover, another limitation is that we cannot determine if these autoantibodies predict risk or not as the study doesn’t have a prospective design.

In conclusion, we found that low levels of IgM autoantibodies against MDA-p210 were associated with occurrence of plaques as well as presence of more and bigger carotid plaques in 64-year-old women. High levels of this autoantibody showed an odds ratio of 0.55 (95% C.I. 0.38–079) for occurrence of any plaque in the carotid arteries, independently of other risk markers and treatment with statins. The observations provide further evidence that immune reactions against epitopes in oxidized LDL are involved in atherosclerosis development by indicating that IgM autoantibodies recognizing such an epitope may have a protective role in carotid atherosclerosis.

## Supporting Information

S1 Table Copy of the questionnaire regarding life style factors, previous and current diseases and medication used by the included patients. (DOCX)

S2 Table Correlations between risk factors for cardiovascular disease and autoantibodies to the apoB-100 peptides p45 and p210 in 594 64-year old women. (DOCX)

S3 Table Autoantibodies to the apoB-100 peptides p45 and p210 in 64-year-old women in relation to treatment with statins. (DOCX)
