# Supplementary material for: Circulating Autoantibodies against the Apolipoprotein B-100 Peptides p45 and p210 in Relation to the Occurrence of Carotid Plaques in 64-Year-Old Women
Source: PLoS One. 2015 Mar 13;10(3):e0120744. doi: 10.1371/journal.pone.0120744 (PMC4358991; doi:10.1371/journal.pone.0120744)
Supplement: S1 Table — (DOCX) [file pone.0120744.s001.docx]

**S1 Table.** Copy of the questionnaire regarding life style factors, previous and current diseases and medication used by the included patients.

**Questionnaire (DIWA study)**

| Date of examination |  |
| --- | --- |
| Name |  |
| Birth date |  |

| Do you smoke? | Yes/no |
| --- | --- |
| If yes, how many cigarettes/dag? |  |
| For how many years have you smoked? |  |
| Have you smoked previously? | Yes/now |
| If yes, when did you quit smoking? |  |
| For how many years did you smoke? |  |
| On average, how many cigarettes did you smoke/day? |  |

| Do you take any medication regularly? | Yes/no |
| --- | --- |
| If yes, please describe which medicines (also vitamines and hormones) |  |

| Have you been healthy? | Yes/no |  |
| --- | --- | --- |
| Check if you have suffered or suffer from any of the following diseases: | | |
| Myocardial infarction | Yes/no | Year? |
| Angina pectoris | Yes/no | Year? |
| Heart failure | Yes/no | Year? |
| Intermittent claudication | Yes/no | Year? |
| Diabetes | Yes/no | Year? |
| High blood pressure | Yes/no | Year? |
| Hyperlipidemia | Yes/no | Year? |
| Stroke | Yes/no | Year? |
| Other | If yes, what disease? | Year? |
