# Supplementary material for: Circulating Autoantibodies against the Apolipoprotein B-100 Peptides p45 and p210 in Relation to the Occurrence of Carotid Plaques in 64-Year-Old Women
Source: PLoS One. 2015 Mar 13;10(3):e0120744. doi: 10.1371/journal.pone.0120744 (PMC4358991; doi:10.1371/journal.pone.0120744)
Supplement: S2 Table — (DOCX) [file pone.0120744.s002.docx]

**S2 Table**. Correlations between risk factors for cardiovascular disease and autoantibodies to the apoB-100 peptides p45 and p210 in 594 64-year old women.

|  | | **SBP** | **DBP** | **HbA1c** | **Fasting blood glucose** | **LDL-cholesterol** | **Apolipoprotein B** | **Apolipoprotein**  **B/A-I** | **C-reactive protein** |
| --- | --- | --- | --- | --- | --- | --- | --- | --- | --- |
| Ab to native p45 | IgG | -0.02 | 0.01 | 0.02 | -0.03 | -0.06 | -0.08* | -0.03 | 0.06 |
|  | IgM | -0.04 | -0.00 | -0.08 | -0.08 | 0.05 | -0.01 | -0.01 | 0.05 |
| Ab to native p210 | IgG | -0.13** | -0.08* | -0.09* | -0.03 | -0.01 | -0.06 | 0.03 | -0.14** |
|  | IgM | -0.10* | -0.08* | -0.04 | -0.01 | 0.03 | -0.04 | 0.03 | -0.02 |
| Ab to MDA-p45 | IgG | -0.06 | 0.00 | 0.01 | 0.00 | -0.05 | -0.07 | 0.03 | 0.03 |
|  | IgM | -0.11** | -0.08 | -0.03 | 0.02 | 0.06 | 0.03 | 0.03 | 0.07 |
| Ab to MDA-p210 | IgG | -0.03 | 0.03 | -0.07 | -0.10* | -0.04 | -0.04 | -0.04 | -0.02 |
|  | IgM | -0.02 | -0.05 | 0.05 | 0.05 | 0.07 | 0.06 | 0.07 | 0.05 |

The Spearman test was used to calculate r values. Ab, antibodies. SBP, systolic blood pressure. DBP, diastolic blood pressure. *p<0.05 **p<0.01
