# Supplementary material for: Circulating Autoantibodies against the Apolipoprotein B-100 Peptides p45 and p210 in Relation to the Occurrence of Carotid Plaques in 64-Year-Old Women
Source: PLoS One. 2015 Mar 13;10(3):e0120744. doi: 10.1371/journal.pone.0120744 (PMC4358991; doi:10.1371/journal.pone.0120744)
Supplement: S3 Table — (DOCX) [file pone.0120744.s003.docx]

**S3 Table.** Autoantibodies to the apoB-100 peptides p45 and p210 in 64-year-old women in relation to treatment with statins.

|  | | **No statin** | **Statin** |
| --- | --- | --- | --- |
| Ab to native p45 | IgG | 0.19 (0.28) | 0.22 (0.29) |
|  | IgM | 0.18 (0.26) | 0.16 (0.20) |
| Ab to native p210 | IgG | 1.17 (0.51) | 1.11 (0.44)* |
|  | IgM | 1.16 (0.58) | 1.04 (0.45)* |
| Ab to MDA-p45 | IgG | 0.81 (0.49) | 0.71 (0.47)* |
|  | IgM | 1.07 (0.69) | 0.94 (0.58)* |
| Ab to MDA-p210 | IgG | 2.43 (0.40) | 2.31 (0.46) |
|  | IgM | 2.30 (0.45) | 2.29 (0.44) |

Ab, antibodies; Values given as absorbance units at 405 nm; Values are median (IQR). *p<0.05
